# Sitting position affects performance in cross-country sit-skiing

**DOI:** 10.1007/s00421-017-3596-y

**Published:** 2017-04-05

**Authors:** M. Lund Ohlsson, M. S. Laaksonen

**Affiliations:** Swedish Winter Sports Research Centre, Department of Health Science, Mid Sweden University, Hus D, 83125 Östersund, Sweden

**Keywords:** Biomechanics, Metabolic rate, Respiratory function, Oxygen uptake

## Abstract

**Purpose:**

In cross-country sit-skiing (XCSS), athletes with reduced trunk control predominantly sit with the knees higher than the hips (KH); a position often associated with large spinal flexion. Therefore, to improve spinal curvature a new sledge with frontal trunk support, where knees are lower than hips (KL) was created. It was hypothesized that the KL position would improve respiratory function and enhance performance in seated double-poling compared to KH.

**Methods:**

Ten female able-bodied cross-country skiers (age 25.5 ± 3.8 years, height 1.65 ± 0.05 m, mass 61.1 ± 6.8 kg) completed a 30 s all-out test (WIN), a submaximal incremental test including 3–7 3 min loads (SUB) and a maximal 3 min time trial (MAX) in both KL and KH positions. During SUB and MAX external power, pole forces, surface electromyography, and kinematics were measured. Metabolic rates were calculated from oxygen consumption and blood lactate concentrations.

**Results:**

KL reduced spinal flexion and range of motion at the hip joint and indicated more muscle activation in the triceps. Performance (W kg^−1^) was impeded in both WIN (KH 1.40 ± 0.30 vs. KL 1.13 ± 0.33, *p* < 0.01) and MAX (KH 0.88 ± 0.19 vs. KL 0.67 ± 0.14, *p* < 0.01). KH resulted in higher gross efficiency (GE) and lower lactate concentration, anaerobic metabolic rate, and minute ventilation for equal power output.

**Conclusions:**

The new KL position can be recommended due to improved respiratory function but may impede performance. Generalization of results to XCSS athletes with reduced trunk muscle control may be limited, but these results can serve as a control for future studies of para-athletes.

## Introduction

Cross-country sit-skiing (XCSS) is an endurance sport (competition time from 3 min to 45–60 min), where athletes propel themselves using a pair of poles while sitting in a sledge which is mounted on a pair of skis. This sport is on the Paralympics agenda and athletes with amputation, spinal cord injuries, cerebral palsy, and growth defects competes against each other in the same event competition. The athletes competing in XCSS can have very different disabilities and are, therefore, divided into locomotor winter (LW) classes 10, 10.5, 11, 11.5, and 12; LW12 athletes have full control and functionality in hip and trunk muscles and full buttock sensibility, whereas LW10 athletes have no control of trunk or hip muscles and no buttock sensibility (IPC Nordic Skiing Classification Rules and Regulations 2015). To make the event competition fair, each class assigned a weight factor depending on how the impairment is assumed to affect performance. Thus, the final result is the product of the athletes’ race time and the weight factor for respective class.

In the 2010 Paralympic Games, it was observed that athletes in XCSS used different sitting positions (Gastaldi et al. 2012). Many LW12 athletes used a “knee seated” position, where the knees were lower than the hips. On the other hand, athletes with highly reduced trunk stability (i.e., LW10, LW10.5) require upper body support from the equipment. This is normally achieved using higher back support from the seat and knees in higher position than hip, (KH). This means that there is a likely association between the low class (LW10, LW10.5) athletes’ control and functionality of trunk and hip muscles and their choice of sitting position in the sledge. The motion of the trunk and its timing in the poling cycle are also related to the athletes’ classification (Rosso et al. 2016); XCSS athletes with less trunk control exhibit less trunk motion and an earlier start of trunk flexion before the peak pole force.

To our knowledge, there are only a few studies that have explored biomechanics and physiology of XCSS. In one of those studies performed with abled bodied athletes (Lajunen 2014), a KH position was less economical [higher oxygen consumption (*V*O_2_) and had higher blood lactate concentration (B-La) and higher minute ventilation (VE)] than a knee seated position with no trunk support. In addition, KH produced higher cycle rate and relative poling time and lower impulse of force and hip range of motion (ROM) (Lajunen 2014). Thus, larger ROM in hip was associated with lower cycle rate (CR) and lower *V*O_2_.

However, athletes with loss of trunk control will not be able to use the knees lower than hip position without support, because this position requires that the athlete can control the trunk muscles. Instead, the KH position might increase the risk of lower back injury because of larger spinal flexion. During spinal flexion, the anterior shear force of the lumbar disc increases (McGill et al. 2000; McGill and Norman 1987). Spinal flexion might also cause forward and downward rotations of the scapula and depression of the acromial process which is related to shoulder pain (Burnham et al. 1993; Samuelsson et al. 2004). The prevalence of lower back injuries in able-bodied XC athletes has been reported higher for classical technique (larger spine flexion) than skating technique (Bahr et al. 2004). From another point of view, excessive spinal flexion may also increase intra-abdominal pressure that in turn may affect the respiratory mechanics (Pelosi et al. 2007).

To improve the posture of the spine by reducing lower back flexion, a new sledge was created for this study. This new sitting position was intended to enable athletes with highly reduced trunk control to use a knee-low position with a frontal trunk support (KL). Therefore, the purpose of this study was to compare the physiology and biomechanics for the new KL position with the KH position in able-bodied athletes. To understand the impact of the equipment without influence of different impairments, this study was performed with able-bodied participants. It was hypothesized that the KL position with improved spinal curvature may improve respiratory function which may consequently improve performance in seated double poling.

## Methods

### Participants

Ten able-bodied healthy female athletes volunteered to participate in the study (mean ± standard deviation (SD), age 25.5 ± 3.8 years, height 1.65 ± 0.05 m, and mass 61.1 ± 6.8 kg). The athletes were competing either in cross-country skiing or biathlon at national senior level at the time of the study. The study was approved by Regional Ethical Review Board in Umeå, Sweden (Dnr 2013-412-31M and Dnr 2015-74-32M), and informed consent was obtained from all individual participants included in the study.

### Overall design

Two familiarization sessions (exercise time 45 min in each) were performed during the week before the main experimental trials. The main experimental trials were two physical tests performed during 2 separate days: one session in each sitting position in randomized order, separated by at least 48 h. Each trial consisted of a 30 s all-out test (WIN), a submaximal incremental test (SUB) and a 3 min time-trial test (MAX). Participants’ body composition was measured using dual-energy X-ray absorptiometry (Lunar iDxa, GE Healthcare) on one morning (after >8 h of fasting) during the test period excluding the trial days.

### Standardization

Participants were asked to perform only easy training session (max 60 min) 1 day before testing and to avoid heavy resistance training for 2 days before the physical tests. In addition, they were asked to eat and drink normally during the day before the trial but to avoid: eating 1 h prior to each trial, alcohol 24 h before, and caffeine on the same day. Drinking during the trials was restricted to water.

### Physical tests

Each trial started with 10 min warm-up including 4 × 5 s maximal intervals. Thereafter, a 30 s all-out test (WIN) was performed. After 60 min passive recovery, participants performed a new warm-up session of 5 min (3 min at wattage of 0.3 × body mass and 2 min of 0.5 × body mass). This was followed by a submaximal incremental test (SUB) including 3–7 submaximal workloads (SUB1–SUB7) of 3 min each with 1 min recovery between the exercise bouts. The respective SUB-level workloads were 15, 22, 30, 37, 45, 52, and 60 W. SUB was terminated when two of the following criteria were fulfilled: respiratory exchange ratio (RER) >1.00, ratings of perceived exertion (RPE) >16, and VE/*V*O_2_ >30. Seven fixed submaximal intensities were used to identify a submaximal level close to B-La 4 mmol/l and RER 1 that would allow for comparison of the biomechanical variables between KL and KH.

The SUB test was followed by a 10 min seated recovery in the sledge before the MAX. In MAX, participants were instructed to ski as far as possible during 3 min and were verbally encouraged during the test. Participants received no real-time feedback during MAX, such as elapsed time, distance covered, or load.

### Measurements and equipment

**Fig. 1:**
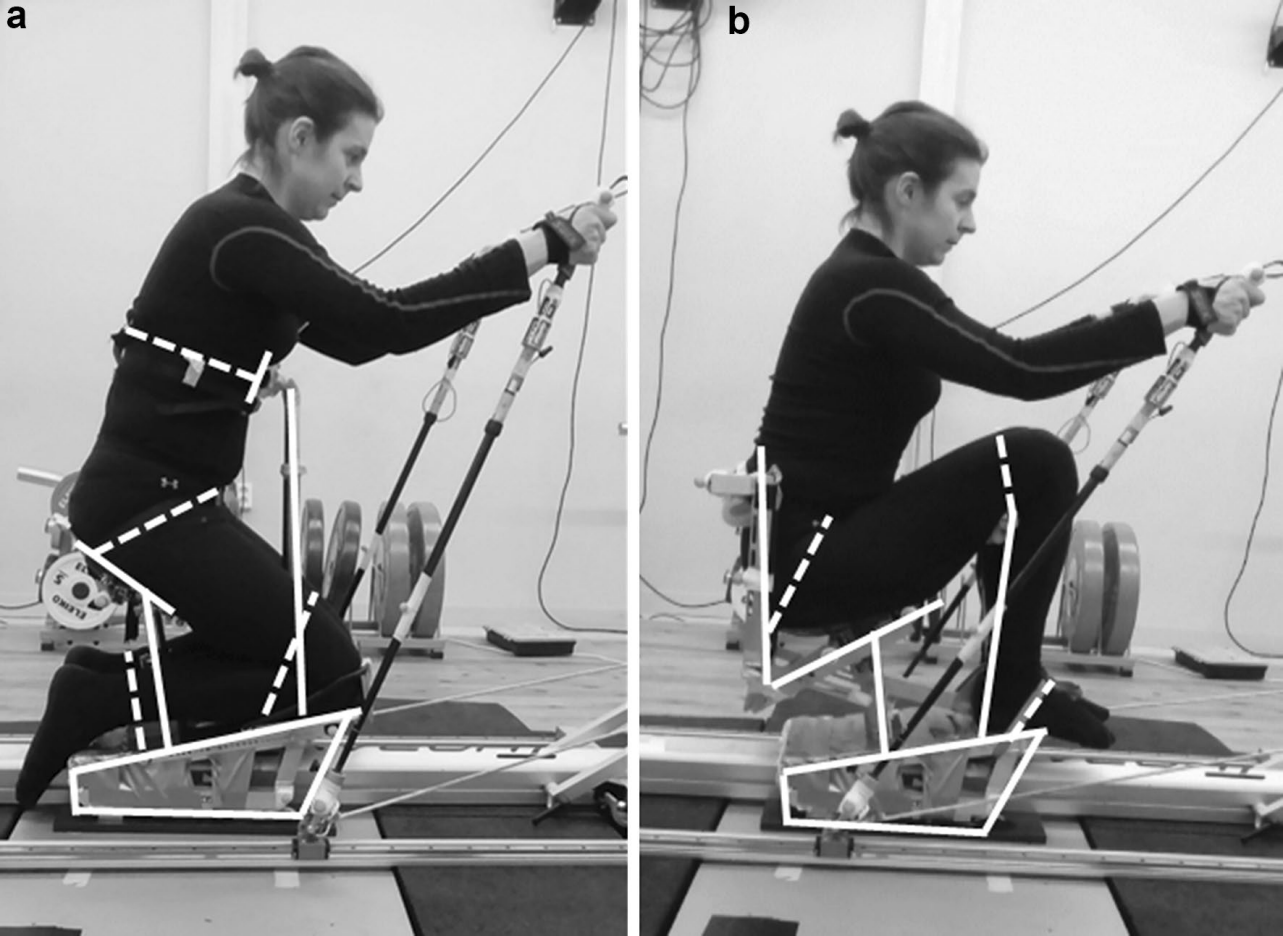
Two different sitting positions **a** knees low and trunk support (KL) and **b** knees high (KH). *Solid lines* indicate fixed structures of the sledge and *dashed lines* the strapping

All physical tests were carried out on a commercial skiing ergometer (ThoraxTrainer, ThoraxTrainer A/S, Kokkedal, Denmark) equipped with two different sledge configurations, KL (Fig. 1a) and KH (Fig. 1b). The participants were strapped to the sledge; in KL around ankles, above knees, around pelvis, and with elastic bands around thorax to the frontal support, and in KH around ankles, knees, and pelvis. In KH, the height of the knees was adjusted as high as possible without pelvis tilting backwards. Vertical sitting height (from bottom of sledge to position of buttocks) was adjusted to 38 cm for KL and 33 cm for KH, which resulted in comparable mean height of center of mass (KL 0.63 ± 0.01 m, KH 0.62 ± 0.01 m, *p* = 0.08).

The respiratory variables during SUB and MAX were monitored breath-by-breath using a stationary metabolimeter (Quark CPET, COSMED, Italy) measuring breathing rate (BR), tidal volume (VT), VE, *V*O_2_, carbon dioxide production (*V*CO_2_), and heart rate (HR). The gas analyzers were calibrated with a mixture of 16.0% O_2_ and 4.5% CO_2_ (Strandmöllen AB, Ljungby, Sweden), and calibration of the flowmeter was performed at low, medium, and high flow rates with a 3 l air syringe (Hans Rudolph, Kansas City, Missouri, USA). Ambient conditions [temperature (20.3 ± 1.1 °C) humidity (30.7 ± 4.4%) and barometric pressure (728.1 ± 6.0 mmHg)] were monitored with an external apparatus (Vaisala PTU 200, Vaisala Oy, Helsinki, Finland).

Ear lobe capillary blood samples were taken immediately after each SUB level and 2 min after the MAX and were used for determination of B-La concentration with a Biosen C-line (EKF diagnostic GmbH, Magdeburg, Germany).

For each cycle, mean power output (PO) and CR were computed from the timing and the moment of inertia of the fly-wheel by the software of the ergometer (ThoraxTrainer ver 1.01, ThoraxTrainer A/S, Kokkedal, Denmark). Strain-gauge force sensors equipped with amplifiers (Biovision, Wehrheim, Germany) were calibrated and used to measure axial pole forces at sampling frequency 250 Hz. The sensors were mounted between the hand grip and the pole. The length of the poles was self-selected by the participants.

Three-dimensional kinematic data were recorded by motion capture with 11 Oqus3+ (Qualisys AB, Gothenburg, Sweden) cameras and the QualysisTrackManager software at a sampling frequency of 200 Hz. A full-body marker set with 36 markers with diameter 12 mm including three on each poles was employed. Joint angles were computed through the kinematical analysis, an optimisation procedure of the over-determinate marker data (Andersen et al. 2010), in the Anybody Modelling system (AMS 6.0, Anybody Technology A/S, Denmark). This was made with a full-body model (available in the AMS model repository, AMMR 1.6.3) together with the poles.

Surface electromyography (EMG) measures were recorded at 1000 Hz by TeleMyo 2400T G2 (Noraxon USA Inc., Scottsdale, USA) and wireless data sent to a receiver (TeleMyo 2400R G2, Noraxon USA Inc., Scottsdale, USA), where data were synchronised with the kinematic and kinetic data. Five muscles were measured on the right side of the body, m. erector spinae longissimus (ES), m. rectus abdominis (RA), m. latissimus dorsi (LD), m. pectoralis major (PM) and m. triceps brachii caput laterale (TRI). Electrodes with 20 mm diameter (Ambu blue sensor N, Ambu A/S, Ballerup, Denmark) were placed with guidance from Surface ElectroMyoGraphy for the Non-Invasive Assessment of Muscles (http://www.seniam.org) for TRI and ES. Placement of the other muscles was: RA—1 cm above the umbilicus and 2 cm lateral to the midline, PM—3 fingers from axilla on the line from axilla to sternum mid when seated and LD 3 fingers from axilla parallel to the edge of scapula when seated and shoulder 90° abducted. Isometric maximum voluntary contractions (MVC) were performed separately for each muscle and in duplicates (5 s each with 2 min recovery time). Positions for MVC were: RA supine position, ES-laying chest down, TRI—sitting with shoulder slightly flexed, elbow flexed 90° and supported from below, PM—bench press with shoulders 90° abducted, LD—pull-up with shoulder flexion 90°, and static pelvis position.

### Data analysis

In a XCSS race, the participants move their body mass around the track by double-poling and gliding. Because this study was performed on a skiing ergometer, i.e., no net forward motion, participants’ performance was defined as the mean PO divided by body mass.

Performance among the participants varied extensively, and in addition, the first SUB levels were very low for some participants. Therefore, the SUB4 was chosen for further biomechanical analysis, B-La and RER approximately 4 mmol l^−1^ and 1, respectively. Right-side kinematics and right pole force were analyzed for four cycles after 120 s for the seven participants completing SUB4. Data were presented as mean of these four cycles. Start of poling cycle was defined when right pole tip was in its foremost position. Poling cycle consisted of poling phase and return phase. Poling phase was defined with start with the pole tips in its foremost position and end in their rearmost position; the return phase was defined the opposite (start in rearmost position and end in foremost position).

Joint angles were defined, in the anatomical position as follows: knee = 0° (flexion positive), hip flexion = 0° (flexion positive), shoulder1 = 0° (flexion positive, extension negative), shoulder2 = 0°, shoulder3 = 0°, elbow = 0° (flexion positive), spine flexion = 0° (angle between pelvis and trunk in sagital plane, kyphosis/flexion negative), and pole angle in sagital plane where horizontal = 0° and pole angle vertical = 90°. Shoulder angles were defined according to International Society of Biomechanics (Wu et al. 2005) with rotation order *x, z, y* (*y*—line directed to the glenohumeral joint from the mid of medial and lateral epicondyles, *z*—perpendicular to the plane formed by glenohumeral joint, lateral and medial epicondyles pointing backwards, *x*—perpendicular to *y* and *z* pointing to the right). Shoulder2 and shoulder3 are the second and third rotation of humerus.

Calibration of strain gauges in the poles was made for 0, 5, 10, 15, and 20 kg to achieve transformation function from voltage to force. The signal was filtered by a 12 Hz low-pass Butterworth filter.

EMG data were processed in Matlab (R2015b, The Mathworks, Inc, Massachusetts, USA), filtered by a Butterworth bandpass filter (50–300 Hz), averaged by root mean square over time window 0.05 s and normalized to MVC. MVC was computed as the maximum voltage over the two trials and instead processed with the time window 0.5 s. Mean cycle EMG was computed for the same four cycles as the kinematics and kinetics. EMG was measured for one participant, and therefore, no statistics were presented.

PO and CR were computed as mean during the whole exercise session, WIN (30 s), each SUB-level (3 min), and MAX (3 min). Mean of respiratory variables was computed in SUB during the third minute and in MAX during the consecutive 25 breaths, where the largest mean *V*O_2_ was observed.

Aerobic metabolic rate (MRae) was computed from *V*O_2_, *V*CO_2_ and gross energy expenditure using RER ≤1.00 according to MRae (W) = (1.1 × RER + 3.9) *V*O_2_ 4148/60 (Weir 1949). Gross efficiency (GE) in SUB was calculated as GE (%) = (PO/MR_ae_) × 100.

The anaerobic metabolic rate (MRan) was computed from B-La by assuming that the positive difference, between B-La after that workload and B-La after SUB1, of 1 mmol/l was equivalent to 3 ml/kg oxygen consumed (di Prampero and Ferretti 1999).

### Statistics

Data were checked for normality using the Shapiro–Wilk analysis. Data were compared pairedwise between the two sitting positions with two-sided paired student *t* tests when normality was observed or with Wilcoxon’s signed rank test in cases, where the assumption of normality was violated.

Two-way repeated-measures analysis of variances (ANOVA) was used to analyze difference in sitting positions in SUB1–SUB4. If Mauchly’s test of sphericity was violated the epsilon was <0.75, the Greenhouse–Geisser correction was applied; while for epsilon >0.75, the Huynh–Feldt correction was used.

Relationships between variables were assessed with Pearson’s correlation analyses. For comparison of linear regressions, an adapted *t* test for linear regressions was used (Zaiontz 2016).

The level of statistical significance was set at α ≤ 0.05. All statistical tests were processed using Office Excel 2013 (Microsoft Corporation, Redmond, Washington, USA) and the Statistical Package for the Social Sciences (SPSS 22, IBM Corp., Armonk, New York, USA). All data were presented as mean ± SD.

## Results

### Performance

**Fig. 2:**
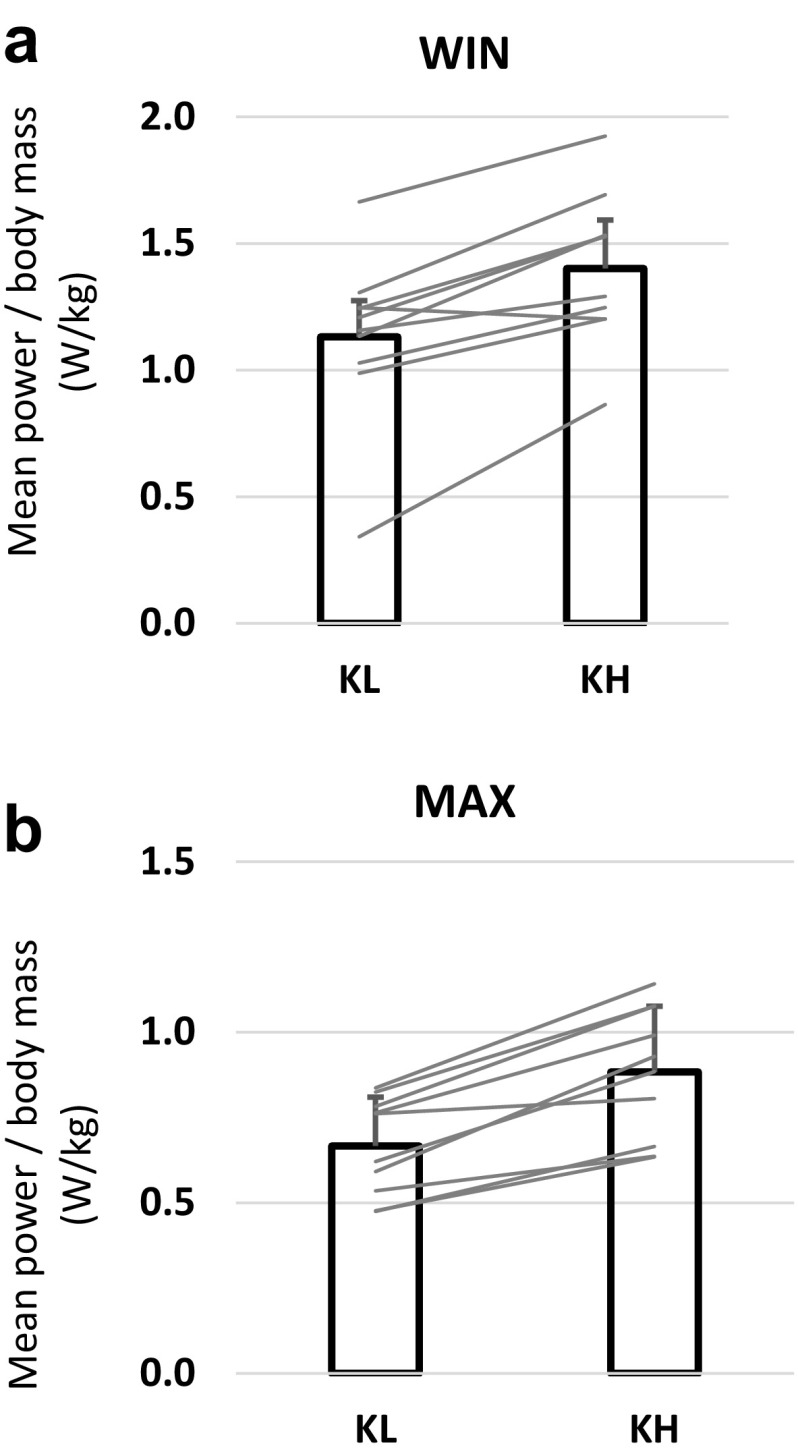
Performance during all-out 30 s test (WIN) (**a**) and maximal time-trial 3 min test (MAX) (**b**) in the two different sitting positions knee-low with frontal trunk support (KL) and knees high (KH). *Error bars* represent standard deviation and *grey lines* indicate individual data for each test

Performance was higher in KH compared to KL both in WIN (1.40 ± 0.30 vs. 1.13 ± 0.33 W/kg, *p* < 0.01, Fig. 2a) and in MAX (0.88 ± 0.19 vs. 0.67 ± 0.14 W/kg, *p* < 0.01, Fig. 2b). There was a correlation between performance (mean power divided with body mass) and ratio of lean mass of arms and trunk to total body mass for both sitting positions (*r* = 0.79 for KH and *r* = 0.90 for KL, *p* < 0.01).

Eight participants completed at least one more SUB level in the KH position compared to KL before stopping criteria were achieved. A number of participants completing each SUB levels (SUB1–SUB7) were for KL = [10, 10, 9, 7, 3, 2, 0] and KH = [9, 10, 10, 8, 7, 4, 2].

### Biomechanics

No difference between the sitting positions was observed neither in cycle time (CT) (KH 1.49 ± 0.03 s and KL 1.43 ± 0.03 s, *p* = 0.39) nor in cycle length (CL) (KH 1.03 ± 0.03 m and KL 1.10 ± 0.03 m, *p* = 0.13) in SUB4. Relative poling time was longer for KL, 50.6 ± 1.2%, compared to KH, 47.5 ± 0.9% (*p* < 0.05). The ratio between CR and BR was 1:1 for both sitting positions.

Kinematics, for selected joint angles presented as maximal (max) minimal (min) and range of motion (ROM), are shown in Table 1. KL resulted in larger knee flexion, hip extension, and reduced spinal flexion, while KH resulted in greater hip ROM and greater spine flexion, both absolute angle and ROM. Smaller Min pole angle and larger ROM for KH were observed, which means that more horizontal poles occur at the end of the poling phase.

**Table 1:** Kinematic data of submaximal workload SUB4 for the seven participants completing this level in both sitting positions

Load	SUB4 (*n* = 7)					
Position	KL			KH		
	Max	Min	ROM	Max	Min	ROM
Knee	139.0 ± 0.007	139.0 ± 0.008	0.04 ± 0.01	125.2 ± 0.2*	124.0 ± 0.08*	1.2 ± 0.01*
Hip flexion	59.9 ± 0.4	53.58 ± 0.5	6.4 ± 0.7	108.2 ± 0.7*	98.4 ± 0.7*	9.8 ± 0.8*
Spine flexion	−16.6 ± 1.1	−30.65 ± 0.8	14.4 ± 1.6	−34.4 ± 1.1*	−57.4 ± 1.2*	23.0 ± 1.7*
Shoulder1	50.4 ± 1.8	−11.12 ± 1.5	61.5 ± 2.6	54.0 ± 2.4	−7.1 ± 2.4	61.1 ± 3.2
Shoulder2	27.8 ± 2.1	−24.70 ± 2.7	52.5 ± 2.4	38.2 ± 3.0	−19.2 ± 2.3	57.4 ± 3.3
Shoulder3	47.5 ± 1.9	19.45 ± 1.5	28.0 ± 2.0	53.0 ± 3.5	21.8 ± 1.3	31.3 ± 3.6
Elbow	107.8 ± 1.9	35.03 ± 2.9	72.8 ± 3.5	107.6 ± 3.2	39.1 ± 2.1	68.5 ± 3.4
Pole angle	68.2 ± 0.9	17.2 ± 0.6	51.1 ± 1.1	69.4 ± 0.8	12.6 ± 0.6*	56.8 ± 1.1*

The table shows selected joint angles (°) for mean of maximal (Max), minimal (Min), and range of motion (ROM) for the two sitting positions KL (knee low) and KH (knee high). The asterisk (*) indicates significant difference (*p* < 0.05) between KL and KH for that variable

Definitions of joint angles, in anatomical position are, knee = 0° (flexion positive), hip = 0° (flexion positive), shoulder1 = 0° (flexion positive, extension negative), shoulder2 = 0°, shoulder3 = 0°, elbow = 0° (flexion positive), spine flexion = 0° (angle between pelvis and trunk in sagital plane, kyphosis/flexion negative), and pole angle in sagital plane where horizontal = 0° and pole angle vertical = 90°

**Fig. 3:**
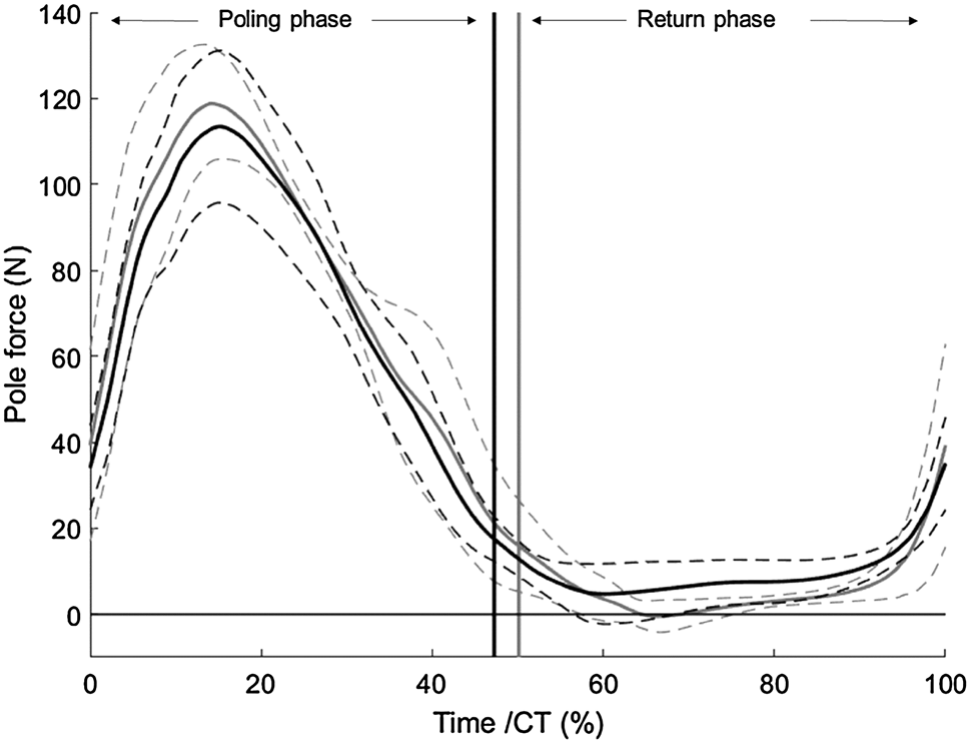
Pole force (SUB4, *n* = 6) plotted against time in percentage of mean cycle time (CT). Position with knees lower than hip (KL) plotted in *grey* and position with knees higher than hip (KH) in *black* (mean force in *solid line* and standard deviation in *dashed line*). *Vertical lines* denotes the end of the poling phase, KH in *black* and KL in *grey*

Pole force profiles for both sitting positions (mean of six participants because one pole force measurement failed) are shown in Fig. 3. There was no difference neither in peak pole forces (KL 120.2 ± 8.2 N vs. KH 115.4 ± 7.4 N, *p* = 0.60) nor in the mean of the pole force profiles over CT (41.1 ± 2.4 vs. 40.2 ± 1.3 N, *p* = 0.81).

**Fig. 4:**
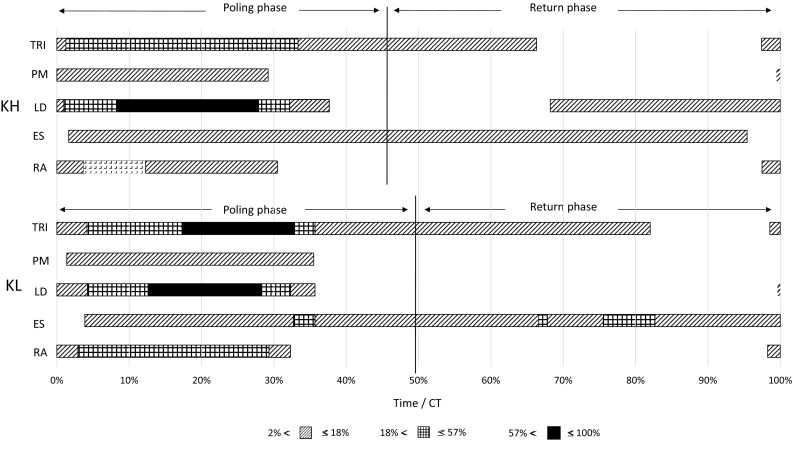
Example of normalized electromyografi (EMG) from one participant for both sitting positions (knees higher than hip, KH, and knees lower than hip, KL) of m. triceps brachii (TRI), m. pectoralis major (PM), m. latissimus dorsi (LD), m. erector spinae (ES), and m. rectus abdominis (RA). Results of one participant, mean of four cycles and presented as percentage of CT. No box means less than or equal to 2% activity, *grey box* more than 2% and less than or equal to 18%, *grid patterned box* more than 18% and less than or equal to 57%, and *filled box* more than 57% and less than or equal to 100%

Normalized EMG pattern of one participant for five muscles, TRI, PM, LD, ES, and RA, in both sitting positions KH and KL (mean of four poling cycles), are shown in Fig. 4. For presentation, the EMG profiles were reduced to blocks of activation as others have done (Holmberg et al. 2005). The results demonstrate earlier onset of TRI, PM, and LD for the KH position but also higher and longer muscle activation for LD as well as lower and shorter activation for TRI lower.

### Physiology

**Fig. 5:**
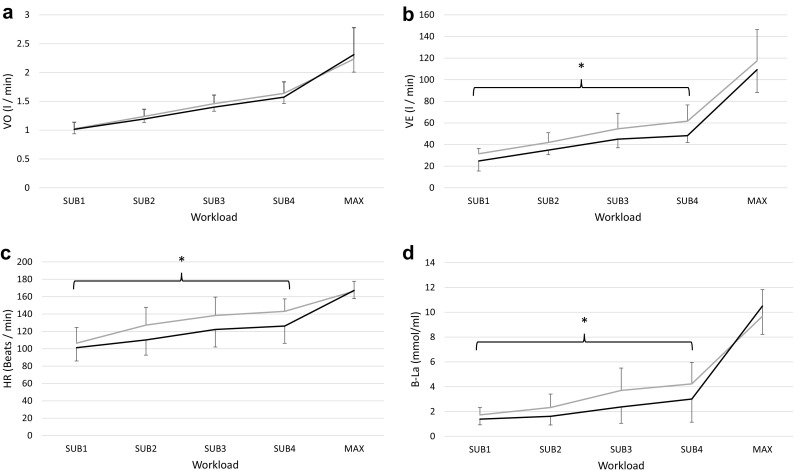
Oxygen uptake (*V*O_2_) (**a**), minute ventilation (VE) (**b**), heart rate (HR) (**c**), and blood lactate (B-La) (**d**) for SUB1-SUB4 and max for sitting position knees low (KL) (*grey line*) and knees high (KH) (*black line*). Data is presented as mean and standard deviation. *Asterisk* (*) denotes main effect of sitting position (*p* < 0.05)

Although there was neither any main effect of position [*F*(1,5) = 0.186, *p* = 0.68] nor any interaction observed between the sitting positions [*F*(3,15) = 2.722, *p* = 0.10] *V*O_2_ was higher for eight of ten participants in KL during SUB (Fig. 5a). In MAX, there was no difference in peak oxygen uptake (*V*O_2Peak_, *p* = 0.48).

Breathing pattern during SUB showed no difference for either tidal volume (VT, l/breath) or BR (breath/min) (Table 2). The product of the two, VE, was significantly higher in the KL position during SUB1-SUB4 [*F*(1,5) = 14.52, *p* < 0.05, Fig. 5b] and the difference increased with increasing workload [interaction effect *F*(3,15) = 6.15, *p* < 0.01].

**Table 2:** Results of physiological parameters at submaximal level 2 (SUB2), 4 (SUB4) and maximal time-trial test (MAX)

Parameter	SUB2 (*n* = 10)		SUB4 (*n* = 7)		MAX (*n* = 10)	
	KL	KH	KL	KH	KL	KH
RER	0.95 ± 0.07	0.89 ± 0.06*	1.02 ± 0.07	0.95 ± 0.04*	1.08 ± 0.08	1.10 ± 0.08
BR (breaths min^−1^)	38.3 ± 6.7	35.1 ± 4.6	43.6 ± 7.8	39.2 ± 5.0	66.6 ± 8.9	67.4 ± 4.6
VT (l breath^−1^)	1.1 ± 0.1	1.0 ± 0.1	1.4 ± 0.2	1.2 ± 0.09	1.8 ± 0.4	1.6 ± 0.3*
MRae (J s^−1^)	426.5 ± 44.9	402.8 ± 21.4	569.6 ± 69.1	537.1 ± 37.1	778.4 ± 190.1	798.6 ± 104.9
MRae/MRtot	0.92 ± 0.06	0.96 ± 0.04*	0.77 ± 0.08	0.86 ± 0.1*	0.61 ± 0.05	0.59 ± 0.05
MRan (J s^−1^)	36.7 ± 30.5	16.8 ± 21.1*	175.9 ± 88.9	99.0 ± 84.1*	507.4 ± 160.0	575.8 ± 147.4
MRan/MRtot	0.074 ± 0.06	0.038 ± 0.05*	0.23 ± 0.08	0.14 ± 0.1*	0.39 ± 0.05	0.41 ± 0.05
MRtot (J s^−1^)	463.2 ± 66.2	419.5 ± 33.8*	745.4 ± 129.6	636.0 ± 108.6*	1285.9 ± 331.7	1374.4 ± 222.2
GE	3.7 ± 0.4	4.1 ± 0.2*	2.7 ± 0.3	3.1 ± 0.2*	–	–

*RER* respiratory exchange ratio, *BR* breathing rate, *VT* tidal volume, *MRae* MRan metabolic rate aerobic and anaerobic and their ratio to MRtot total metabolic rate (MRae + MRan), *GE* gross efficiency

Asterisk (*) denotes significant difference (*p* < 0.05) between knee low (KL) and knee high (KH) for that workload

**Fig. 6:**
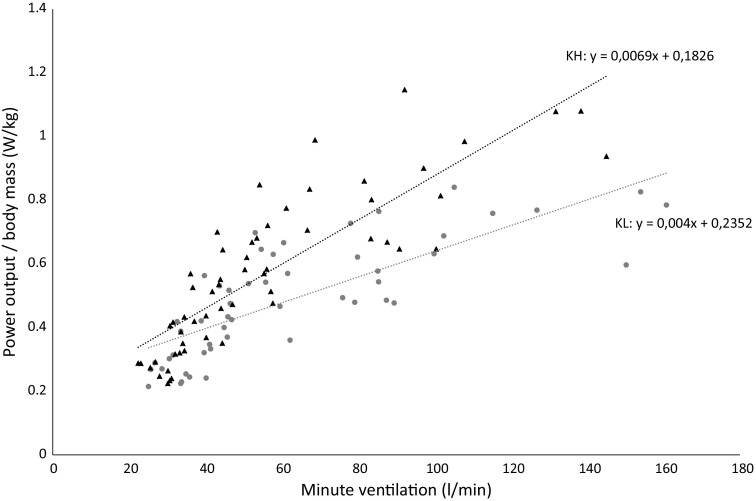
Correlation and linear regression of workloads and minute ventilation (VE) in SUB and MAX for both sitting positions. Sitting position knee-low with frontal trunk support (KL *grey circles*) and knees high (KH *black* triangles). Significant difference of the linear regressions (*p* < 0.001) between the sitting positions

During MAX, VT was higher in KL (*p* < 0.05), while BR was similar between KL and KH. Linear regression of VE in comparison to workload/body mass (in both SUB and MAX) revealed positive correlations for KL (*r* = 0.85, *p* < 0.01) and for KH (*r* = 0.78, *p* < 0.01) (Fig. 6). The regression lines were significantly different (*p* < 0.001), showing that higher VE were utilized in position KL for the same workload.

HR was lower for KH during the SUB1–SUB4 [main effect *F*(1,5) = 9.39, *p* < 0.05] but showed no difference in MAX (*p* = 0.79) (Fig. 5c). MRae was similar between KH and KL [*F*(1,5) = 1.099, *p* = 0.34], but there was a trend towards interaction between sitting positions; higher MRae in KL when workload increased [*F*(3,15) = 3.20, *p* = 0.05] (Table 2). There was a main effect of position on both B-La and MRan, i.e., higher in position KL [*F*(1,5) = 17.71, *p* < 0.01 and *F*(1,5) = 28.08, *p* < 0.01, respectively, Fig. 5d and Table 2]. The influence of sitting position on anaerobic metabolism (B-La and MRan) was higher with higher workloads in SUB [interaction effect *F*(1.08,5.38) = 14.13, *p* < 0.05, and *F*(1.10, 6.59) = 26.58, *p* < 0.01, respectively]. In MAX, no significant difference was observed (*p* = 0.05, *p* = 0.05).

RER was higher in KL than KH during SUB [main effect of position *F*(1,5) = 22.42, *p* < 0.01] but showed no difference during MAX (Table 2). During SUB, GE was between 2.7–4.1% and decreased with increasing workload. Higher GE was observed for KH in SUB2-4 [main effect of position *F*(1,6) = 7.46, *p* < 0.05, Table 2]. Higher total metabolic rate (MRtot) was observed for KL [*F*(1,5) = 10.27, *p* < 0.05] and showed no difference in MAX (*p* = 0.15) which resulted in higher efficiency (PO/MRtot) in MAX for KH (3.9 ± 0.7 vs. 3.2 ± 0.6%, *p* < 0.05).

## Discussion

The purpose of this study was to compare a KL position with frontal trunk support with the KH position using biomechanical and physiological measures. The KL position was created to reduce spine flexion which was confirmed but as well as to improve respiratory function which may enhance performance in seated double poling. The main findings of the present study were that for able-bodied participants, the KL position showed higher ventilation (i.e., improved respiratory function in terms of VT and VE), but performance was impeded.

In the KL position, the trunk was fixed to the frontal support by elastic bands. Instead in the KH position, there was no strapping of spine to the backrest instead the passive resistance of back and hip extensors was intended to hinder spine flexion. The knees were positioned as high as possible without posterior tilt of pelvis. XCSS athletes in low classes (e.g., LW10, LW10.5) using a KH position either have a strap around the upper abdominals to the backrest or uses the passive resistance of their back and hip extensors when hips are flexed to restrict forward motion. The hip–spine flexion–extension is small for those athletes (Karczewska-Lindinger et al. 2016) and sometimes also a hip–spine extension occurs during the poling phase, which is opposite to able-bodied double-poling and higher classes (e.g. LW12) in XCSS who utilize hip–spine flexion during poling-phase. Because there was no strapping of spine in KH and as able-bodied participants were used the hip–trunk motion might have been different than low class athletes in XCSS.

Due to the fixation of the trunk, the position of the shoulder was more still in KL. This also implied less motion in the hip joints, and the spine curvature was more constant (lower ROM). The spine was in less flexion (less kyphotic) which is better for spine (Pope et al. 2002) and shoulder loading (Samuelsson et al. 2004). The sitting position also affected the motion of the pole, which had smaller ROM because of less horizontal pole in the end of the poling phase during KL. However, the results showed no significant difference in CT or CL between sitting positions, although KL had longer relative poling time. In able-bodied XC double-poling (standing position), Holmberg et al. (2005) have shown that shorter relative poling phase (which enhanced recovery time between the pole strokes) together with higher peak pole force, is associated with faster skiing speed.

The KH position was associated with larger ROM in hip and spine which will lead to higher linear and angular momentum of the trunk. If the timing is right, this motion of the trunk can increase both performance and efficiency because of reduced muscular activity. We observed that normalized EMG was higher in TRI and lower in LD during KL than KH, which indicates more arm activity than shoulder activity. The trunk muscles, RA and ES, also had earlier onset and slightly lower activity in the KL position. Therefore, the KH position is likely to distribute the muscle work to larger part of the body, whereas in the KL position, the work relies relatively more on the arm muscles. We will, however, highlight that this observation was obtained only for one participant. It is also a drawback of using able-bodied participants as the KH position was intended to imitate the sitting position for low class XCSS athletes with highly reduced trunk control. The current study indicates that power is produced in the trunk, and perhaps, there is also higher trunk power in the KH position compared to the KL position.

It is known in standing XC that athletes have exceptionally high maximal *V*O_2Peak_ and that this is one of the most important factors for performance (Holmberg 2015). It was assumed that *V*O_2Peak_ in the present study was lower compared to standing skiing due to smaller relative active muscle mass during exercise. *V*O_2Peak_ values observed in the present study were, however, comparable to other studies conducted with XCSS athletes. A study of 14 Italian male (9 spinal cord injured, 3 lower limb amputee, 1 poliomyelitis) showed *V*O_2Peak_ values of approximately 2.9 l min (Bernardi et al. 2012). The slightly lower values in the present study can be explained by female participants.

While *V*O_2_ and MRae were similar at both SUB and MAX between sitting positions, GE was lower in KL. The GE was relatively low (2–6%) compared to wheel–chair propulsion (2–10%) (Woude et al. 1986), arm cranking (5–15%) (Powers et al. 1984), and arm cycling (12–25%) (Verellen et al. 2012). Many arm and leg cycling studies report lower gross efficiency for arm cycling than leg cycling, for references (Sawka 1986), and higher gross efficiency for whole body exercise (Holmberg et al. 2006). The gross efficiency was decreased when increasing exercise intensity; this has also been observed in standing double poling (Andersson et al. 2016). One reason can be that the mechanical efficiency decreases when the range of motion of the limbs is large when increasing exercise intensity.

It was demonstrated with one para-athlete tested in similar KL and KH positions as the present study (but both with frontal trunk support) that KL position was more economical during submaximal exercise than KH (Hofmann et al. 2016). In that study, both positions had fixed trunk, and therefore, the exercise was likely more arm-powered. In addition, the KH position had shorter CT compared to the current study.

Lajunen and colleagues (2014) compared economy in two sitting positions for able-bodied XC athletes. One position was similar to the KH position in the current study and the other position was knee seated with knees below the hips and trunk without any support. KH had more restricted trunk motion compared to KL without trunk support, and hence, muscles in trunk and hip were working more. They also showed as in the present study that the sitting position using larger part of the body had higher gross efficiency, lower lactate concentration, and lower VE.

One study using hand-biking has also compared an arm-powered and arm + trunk-powered posture (Verellen et al. 2012). That particular study showed that peak PO, *V*O_2Peak,_ and peak VE were lower and blood lactate and GE were higher in the arm-powered position. In contrast, the present study observed that GE and VE were lower in arm-powered KL position. Different GE between the studies can be explained by differences in study populations and their ability for trunk power contribution. Verellen et al. (2012) had participants with no prior experience, whereas in the present study, the participants where national class XC skiers. In addition, in the present study, GE was higher in arm and trunk-powered position (KH), where the use of trunk in a more effective way reduces the work in the arms.

The present study showed that the B-La and the MRan were higher in KL during SUB. One reason can be that the KL position had higher levels of activation for arm muscles which may have led to higher rates of anaerobic energy metabolism. This is supported by observations that arm muscles have a higher percentage of type II fibers (Koppo et al. 2002; Pendergast 1989). By assuming that the arms have less oxidative capacity, anaerobic threshold will occur at a lower *V*O_2_ (Sawka 1986). Both slow and fast components of *V*O_2_ kinetics are shown to be slower, in arm cycling compared to leg cycling (Koppo et al. 2002), which is consistent with a greater or earlier recruitment of type II fibers. However, the *V*O_2_ kinetics are trainable because metabolism during arm exercise has been shown to differ between trained and untrained individuals (Pendergast 1989). Arm-trained individuals have higher oxidative capacity in the arms and faster *V*O_2_ response, and thus lower lactic acid accumulation (Pendergast 1989). In MAX, the performance was higher for KH probably because the muscular work was distributed over larger part of the body. The greater mass of working muscles in KH also produced higher lactate concentration and hence resulted in higher MRan in MAX.

There might also be circulatory factors behind the differences in the energy metabolism and VE. The current study showed higher HR in KL in SUB but no difference in MAX. In maximal time-trial arm cycling elicits, lower peak heart rate and *V*O_2_ (Zinner et al. 2016) and lower peak stroke volume which implies lower cardiac output (Calbet et al. 2015) compared to leg cycling. In addition, mean blood pressure has also been observed to be higher in arm cycling compared to leg cycling (Calbet et al. 2015). An increased sympathetic response in arm cycling gives a higher vasoconstrictor tone in non-active muscles and hence larger resistance in the vessels (Sawka 1986). Both active muscle mass normalized vascular conductance and fractional oxygen extraction are lower during arm exercise than leg or whole body exercise (Calbet et al. 2015). This is compensated for in arm cycling by a higher perfusion pressure to increase oxygen delivery to the arms. Thus, it can be speculated that KL induces higher blood and perfusion pressure.

The present study showed that VE was higher in KL at the same workload. The higher anaerobic metabolic rate in KL could be potential reason for the higher VE observed in the present study. It has been shown that arm cycling is associated with higher VE and B-La and lower arterial pH, partial pressure of carbon dioxide, and arterial bicarbonate concentration compared to whole body cycling (Sawka 1986). This observation agrees with the results of the current study, where higher VE and blood lactate were observed in the KL position and less muscle mass utilized.

The anaerobic energy contribution in the current study was estimated from increases in peak lactate concentration which has some drawbacks. Lactate concentration in a sample as in this study reflects the balance between production/release and uptake/utilization by the active muscles. It has been shown in standing XC that the arms produce more lactate than they can utilize while the legs oxidize most of the lactate produced by the whole body (Van Hall et al. 2003). The current study examined the difference between two positions where the legs were strapped and kept static. Because the hip and trunk motion differed slightly between the sitting positions, the ability of the legs to uptake and utilize lactate may have been affected.

In the current study, no difference between the sitting positions were observed in CR which implies similar contraction frequency. This is an important factor when comparing motion techniques, because contraction frequency affects muscle blood flow and oxygen uptake (Ferguson et al. 2001), i.e., higher frequency is associated with higher *V*O_2_. It has also been shown that skeletal muscle blood flow during dynamic muscle contractions is higher compared to isometric (static) muscle work (Laaksonen et al. 2003). In addition, dynamical high frequency contractions or isometric contractions especially in the upper body rise the blood pressure (Sawka 1986), and hence, the whole body vascular conductance is lowered.

This new KL position was created for para-athletes with reduced trunk muscle control. In the present study, the able-bodied athlete participants likely performed differently than para-athletes due to differences in muscular control, muscular strength, and vasoconstriction. When participants with reduced trunk muscle control were tested the trunk kinematics and pole forces differed (Rosso et al. 2016). Athletes with spinal cord injury above vertebrae Th6 experience impaired trunk muscle control but also limited maximal heart rate owing to a lack of sympathetic drive to the heart. In addition, this affects blood distribution in the body which reduces venous return, limits cardiac stroke volume during exercise, and hence influences performance (Theisen 2012).

Using laboratory studies to mimic field performance always includes drawbacks. To our knowledge, there is no study which has investigated how the thorax trainer mimics natural skiing. However, another double-poling ergometer (Concept2 Inc, Morrisville, Vermont, USA) has shown good agreement for XCSS athletes with natural skiing in incline 2.5° for speed, pole force, and EMG (Rosso et al. 2017).

## Conclusions

The new KL position with frontal trunk support was created, because it was hypothesized not only to enhance the performance but also to improve body posture compared to the KH position. This study showed that the flexion of the spine was reduced in the KL position which thereby may decrease the risk of injuries. The KL position was also associated with improved respiration, but it impeded performance and efficiency. Carefully, it can be speculated that these are affected by less compression of the abdomen and more isolated muscle power contribution, i.e., from arms and shoulders. This study was performed on able-bodied athletes and thereby enabled trunk muscle power contribution, especially in the KH position where the trunk motion was less restricted. Generalization of the present results to XCSS athletes with reduced trunk muscle control may be limited, but these results can serve as a control for future studies of para-athletes.

## Abbreviations

ANOVA: Analysis of variances
B-La: Blood lactate concentration
BR: Breathing rate
XCSS: Cross-country sit-skiing
XC: Cross-country skiing
CR: Cycle rate
CL: Cycle length
CT: Cycle time
GE: Gross efficiency
HR: Heart rate
KH: Knees higher than hips
KL: Knees lower than hips with a frontal trunk support
LW: Locomotor winter
MAX: Maximal time-trial test
Max: Mean of maximal joint angle
Min: Mean of minimal joint angle
MRae: Metabolic rate aerobic
MRan: Metabolic rate anaerobic
MRtot: Total metabolic rate
PO: Power output
RER: Respiratory exchange ratio
ROM: Range of motion
SUB: Submaximal incremental test
*V*O_2_: Oxygen uptake
*V*O_2Peak_: Oxygen uptake peak
*V*CO_2_: Carbon dioxide production
VT: Tidal volume
VE: Minute ventilation
WIN: All-out 30 s test
